# The Essentials of Medical Chemistry and Urinalysis

**Published:** 1888-07

**Authors:** 


**The Essentials of Medical Chemistry and Urinalysis.** By S. E. WOODY, A. M., M. D., Prof. of Chemistry and Public Hygiene, etc., in the Kentucky School of Medicine. Second edition. Louisville: J. P. Morton & Co.

This little book is one of the best compends of chemistry we have yet seen. The author has kept the medical bearings of chemistry constantly in view, and it is not lumbered up with a mass of facts which serve only to confuse the student without instructing him. The book is well printed and excellently illustrated. The medical student will find this work a valuable aid in acquiring the principles of what is usually considered the dullest of the elementary medical sciences.

